# Quantitative proteomics analysis reveals the key proteins related to semen quality in Niangya yaks

**DOI:** 10.1186/s12953-023-00222-9

**Published:** 2023-10-24

**Authors:** Yaomei Wang, Yuchao Liu, Tingting Cao, Chunyuan Shi, Zili Ren, Yanling Zhao

**Affiliations:** Tibet Agricultural and Animal Husbandry University, Linzhi, Tibet, 860000 P.R. China

**Keywords:** Niangya yaks, Semen quality, Comparative proteomics, PRM, Differentially expressed proteins

## Abstract

**Background:**

Proteins related to sperm motility and sperm morphology have an important impact on sperm function such as metabolism, motility and fertilisation etc. An understanding of the key proteins related to semen quality in Niangya yaks would help to provide support for breeding. However, the key proteins that affect semen quality in Niangya yaks remain unclear.

**Methods:**

Herein, we applied tandem mass tag (TMT) labeling and liquid chromatography-tandem mass spectrometry (LC‒MS/MS) to analyze the expression levels of sperm proteins in groups of high- and low-quality semen from Niangya yaks. And fifteen differentially expressed proteins (DEPs) were randomly selected for expression level validation by parallel reaction monitoring (PRM).

**Results:**

Of the 2,092 quantified proteins, 280 were identified as DEPs in the high-quality group versus the low-quality group. Gene Ontology (GO) analysis revealed that in terms of biological pathways, the DEPs were mainly involved in metabolic processes, cell transformation processes, and single organism metabolic processes. In terms of cell composition, the DEPs were mainly located in the cell membrane, organelle, molecular complex. In terms of molecular functions, the most abundant functions of the DEPs were catalytic activity, binding activity, transport activity, and enzyme regulation activity. Kyoto Encyclopedia of Genes and Genomes (KEGG) analysis revealed that the DEPs were mainly involved in the cytokine and cytokine receptor interaction, notch signaling pathway, lysine biosynthesis, renal function-related protein and proteasome pathway. From protein-protein interaction (PPI) analysis of DEPs involved in important pathways, 6 related proteins affecting the semen quality of Niangya yaks were identified. And the results of the PRM and TMT analysis were consistent.

**Conclusions:**

The differential sperm proteomic analysis of high- and low-quality semen from Niangya yaks, revealed 6 proteins (PSMC5, PSMD8, PSMB3, HSP90AA1, UGP2 and HSPB1), were mainly concentrated in energy production and metabolism, might play important roles in semen quality, which could serve as candidates for the selection and breeding of Niangya yaks.

**Supplementary Information:**

The online version contains supplementary material available at 10.1186/s12953-023-00222-9.

## Background

Yaks can adapt well to the environment of the Qinghai-Tibet Plateau [1] and are the main economic source of the local people [2]. The Niangya yak, a Xizang Naqu City Jiali County specialty, is an excellent breed of yak in Tibet and is favored by farmers and herdsmen for its fresh, tender meat and delicious taste. Breeding this breed is an effective way to improve the production performance of Niangya yaks and prevent breed degradation. Promoting artificial insemination technology in the Qinghai-Tibet Plateau and strengthening research on yak sperm-related protein levels are the key factors for breed improvement and production performance improvement of Niangya yaks, as well as for promoting the development of the local economy [3]. The proteome of bovine spermatozoa consists of hundreds of proteins, and although most of their functions are still unclear, studies have shown that most of them are related to fertilization ability [4]. The absence, presence, underexpression, or overexpression of specific proteins may alter sperm function, reduce fertilization ability, and thus reduce the ability of sperm to function in reproduction [5]. The proteomes of sperm and seminal plasma of bulls with different levels of reproductive performance were compared to provide a reference for improving the reproductive ability of Holstein cattle with low reproductive performance [6]. It was identified that the proteins related to buffalo fertility from the comparisons of the proteomes of sperm from buffalo with high and low fecundity [7]. Wu [8] adopted iTRAQ to analyze the difference of muscle fiber composition, metabolic and protein markers between the longissimus dorsi muscle of yak and cattle, and identification 12 proteins correlated with meat quality traits. Proteins associated with horn development in yaks [9] and proteomic analysis of testicles of Tianzhu white yaks at different sexual development stages have been performed [10]. A proteomics study of testicles of adult yaks and intersexual yaks used two-dimensional electrophoresis technology [11]. Selection and breeding of this population is an effective way to improve the production and reproduction performance of Niangya yaks and this prevents the breed degradation. Obviously, it can identify the proteins related to semen quality in Niangya yaks to analyze the expression levels of sperm proteins in groups of high- and low-quality semen from Niangya yaks by comparative proteomics, which would help to the breeding of Niangya yaks. However, the key proteins that affect the quality of semen from Niangya yaks remain unclear.

Here, in this study, tandem mass tag (TMT) quantitative sperm proteomic analysis of a high-quality group (H) and a low-quality group (L) was used to identify the proteins related to semen quality in Niangya yaks, and 15 differentially expressed proteins (DEPs) were randomly selected for quantitative validation analysis through parallel reaction monitoring (PRM) technology. The present study results provide a support for the selection and breeding of Niangya yaks.

## Methods

### Semen collection and quality analysis

Fresh semen samples were obtained from 6 healthy male Niangya yaks (3 each in high-quality and low-quality groups) aged between 4.5-6.5 years and proven to be fertile maintained at the Dangxiong yak frozen semen station (Dangxiong County, Lhasa City) using an artificial vagina.

In the present study, semen quality parameters include semen volume, sperm concentration, sperm motility and sperm abnormality were analyzed. Semen volume was directly measured with a graduated collecting tube, and the other parameters were analyzed using a CASA system (Hamilton Thorne Biosciences, MA) according to the manufacturer’s instructions. In short, after the original semen was diluted 10 times with same temperature diluent, a drop of the semen was dropped onto a preheated (37 °C) glass slide and was coverd by clean covered glass. At least 3 visual felds were observed to obtain an average. The rate of sperm abnormality was determined by modified Papanicolaou staining [12]. Semen quality parameters were analyzed by IBM SPSS19.0 software and divided into two groups of high- and low-quality with significant differences (incorporate the test of significance - t test).

### Sperm protein preparation

The semen samples were centrifuged at 2,000 g for 5 min, and the supernatant was removed, then the sperm pellets were washed three times with cold phosphate-buffered saline (PBS) to remove impurities. The sperm pellets were resuspended in lysis buffer (8 M urea, 4% CHAPS, 50 mM DTT and protease inhibitor, pH 8.0) at 4 °C. The lysates were centrifuged at 10,000 g for 30 min to remove insoluble material, and the supernatants were collected, which contained the sperm protein [13]. The protein concentration was measured with a Bradford protein assay kit (P0006C, Beyotime Institute of Biotechnology, Nanjing, China). The protein quality was assessed using SDS-PAGE (5% stacking gel, 12% separating gel), and the gel was stained by Coomassie Blue R-250 for visualization of the protein bands.

### TMT analysis

The qualified sperm proteins from the three samples in the high-quality group (H) and the three samples in the low-quality group (L) were separately pooled in equal parts as a biological sample, then each biological sample was separately made into three parallel mixed samples. Equal amount (100 µg each) of protein from the six samples (three H group and three L group) were digested using filter-assisted sample preparation (FASP) as previously described [14]. The resulting peptides of the H and L groups were subsequently labeled 126 (H1), 127 (H2), 128 (H3), 129 (L1), 130 (L2), and 131 (L3) according to the manufacturer’s instructions (TMT Sixplex Isobaric Mass Tagging Kit; Thermo Fisher Scientific, Rockford, IL, USA, Cat. No. 90064), pooled, dried by centrifugal evaporation and redissolved in mobile phase A (2% acetonitrile (ACN), pH 10.0). The peptides were further fractionated using a C18 column (4.6 × 250 mm, 5 μm) on an L3000 HPLC (Rigol, China). The retained peptides were eluted with mobile phase A and mobile phase B (98% ACN, pH 10.0) at a flow rate of 700 µL/min. Thirty-six fractions were collected and combined to make 6 fractions and reduce peptide complexity, according to the protein properties. The fractionated peptides were desalted using Zip-Tip C18 Tips (Millipore, USA; Cat. No. 87782), redissolved in buffer A (2% acetonitrile, 0.1% formic acid) and centrifuged at 12,000 rpm for 10 min. Ten microliters of the supernatants were injected into the nano UPLC‒MS/MS system consisting of a Nanoflow HPLC system (EASY-nLC 1000 system from Thermo Scientific) and Orbitrap Fusion Lumos mass spectrometer (Thermo Scientific). The sample was loaded onto an Acclaim PepMap100 C18 column and then separated by an EASY-Spray C18 column. The mass spectrometer was operated in positive ion mode (source voltage 2.1 KV), and the full MS scans were performed in the Orbitrap over the range of 300-1,500 m/z at a resolution of 120,000. For MS/MS scans, the 20 most abundant ions with multiple charge states were selected for higher energy collisional dissociation fragmentation following one MS full scan. Independent samples t test was used to compare differences of protein expression between the H and L groups and calculate *P* values. Fold change of ≥ 1.3 or ≤ 0.77 was set as the threshold to identify DEPs.

### Database search and bioinformatics

The obtained spectra were searched against UniProt _ Bos grunniens (2021.08.09 download). Fasta database for peptide identification and quantification using PD2.4 software. The search parameters were specified as follows: two missed cleavage sites were allowed, the mass tolerance was set at 10 ppm for precursor ions and ± 0.02 Da for fragment ions, carbamidomethylation was set as a static modification, and M oxidation and TMT-6plex were set as dynamic modifications. The false-positive detection rate (FDR) was calculated using a decoy database search with an FDR < 1.0%. A *P* < 0.05 and a fold change (FC) ≥ 1.3 or ≤ 0.77 were set as the thresholds to identify differentially expressed proteins (DEPs) (i.e., up- or downregulated proteins). The DEPs were analyzed and mapped by Gene Ontology (GO) and Kyoto Encyclopedia of Genes and Genomes (KEGG) (https://international.biocloud.net/zh/dashboard) [15]. PPI networks were generated and analyzed using STRING online software (https://string-db.org/) [16].

### Validation of protein expression levels by PRM

Among the 280 DEPs, 15 proteins were randomly selected, and PRM technology was used to verify the accuracy of the protein expression level and protein function obtained by TMT labeling quantitative proteomics analysis. Signature peptides for the target proteins were defined according to the TMT data, and only unique peptide sequences were selected for PRM analysis. The proteins (60 µg) were prepared, reduced, alkylated, and digested with trypsin following the protocol for TMT analysis. The obtained peptide mixtures were introduced into the mass spectrometer via a C18 trap column (0.10 × 20 mm; 3 μm) and then via a C18 column (0.15 × 120 mm; 1.9 μm).

The MS measurement was performed using a quadrupole mass filter-equipped bench-top Orbitrap mass spectrometer (Q-Exactive; Thermo Scientific). Relevant ion information used for PRM analysis in Additional file 1: Table S1. The raw data obtained were then analyzed using Proteome Discoverer 1.4 (Thermo Fisher Scientific). The FDR was set to 0.01 for proteins and peptides. Skyline 2.6 software was used for quantitative data processing and proteomic analysis. Three biological replicates were included in each group (H and L) in the PRM analysis.

### Statistical analysis

Statistical analyses were performed using IBM SPSS Statistics version 19.0 (SPSS Inc., Chicago, USA). Sperm motility parameters and the proteomic data were analysed with significant differences between the two groups of samples was tested by independent samples t test. All quantitative data are presented as the mean ± standard deviation (S.D.). We considered *P* < 0.05 (*) statistically significant and *P* < 0.01 (**) extremely statistically significant.

## Results

### Semen quality analysis

As shown in Table 1, the semen volume, sperm concentration and sperm motility of the H group were highly significantly higher than those of the L group. Sperm abnormalities in the H group were highly significantly lower than those in the L group. Visibility, semen quality in the H group were significantly superior than those in the L group.

**Table 1:** Semen analysis from high- and low- quality groups of Niangya yaks

Semen	High-quality group (H)	Low-quality group (L)	*P* value
Semen volume (mL)	5.05 ± 0.09**	4.22 ± 0.30	0.01
Sperm concentration (10^8^/mL)	11.50 ± 0.55**	10.07 ± 0.06	0.01
Sperm motility	0.80 ± 0.01**	0.71 ± 0.03	0.00
Sperm abnormality (%)	12.30 ± 0.26	15.24 ± 0.11**	0.00

H = High-quality group (*n* = 3), L = Low-quality group (*n* = 3)

Each bar represents the mean ± standard deviation (S.D.)

**Highly signifcant difference (*P* < 0.01)

### Protein identification and analysis of DEPs

**Fig. 1:**
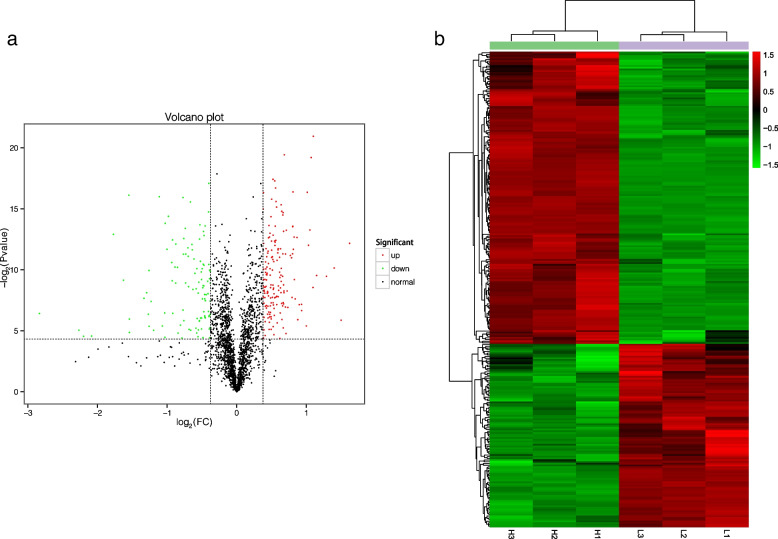
Quality control results of the proteins and differentially expressed protein (DEPs) identification. **a** Volcano plot of differentially expressed proteins. **b** Cluster analysis of differential protein expression (in the figure, each column represents a cluster of different samples, and each row represents a cluster of different proteins). Clustering was determined by the log_2_ value of expression, and the expression of proteins are shown in red (high expression protein) and green (low expression protein)

The obtained protein bands were clear and uniform, indicating that these protein samples were qualified (Additional file 2: Fig. S1). In this study, a total of 2165 proteins were identified successfully, 2092 of them were labeled, indicating the labeled efficiency of 96.6%. Spectral Peak intensity was normalized for quantitation by Scaffold software. Among the 2092 proteins (Additional file 3: Table S2), 280 DEPs were identified to have a 1.3-fold change threshold (*P* < 0.05), of which 172 proteins in the high-quality group were upregulated and 108 proteins were downregulated (Fig. 1a and Additional file 4: Table S3). Cluster analysis based on the protein abundance data of the 280 DEPs showed that the three biological duplications in each group were clustered into one group. That is, H1, H2, and H3 (high-quality group) were clustered into one group, and L1, L2, and L3 (low-quality group) were clustered into one group (Fig. 1b).

### GO and KEGG analyses of DEPs

**Fig. 2:**
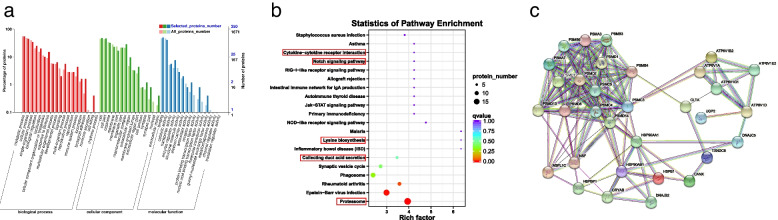
GO, KEGG analyses and protein-protein interaction (PPI) network construction of DEPs. **a** GO analysis of differentially expressed proteins in Niangya yak sperm (biological process, cell component, molecular function). **b** Scatter map of KEGG pathway enrichment of differentially expressed proteins. (Circle: pathway. Abscissa: enrichment factor. Ordinate: pathway name. Enrichment factor: the ratio of the proportion of annotated differentially expressed proteins in a protein pathway to the proportion of all annotated proteins in that pathway. Enrichment factor size: the level at which a protein is enriched in the pathway; the larger the enrichment factor, the more significant the enrichment level of differentially expressed proteins in this pathway. Color of the circle: qvalue, where qvalue is the *P* value after correction for multiple hypothesis testing, and a smaller qvalue indicates a more reliable enrichment significance of the differentially expressed proteins in this pathway. Circle size: the number of proteins enriched within the pathway, with larger circles indicating more proteins. This figure is from www.kegg.jp/kegg/kegg1.html) **c** PPI network of the DEPs involved in important pathways. (The nodes are proteins, and the edges are interactions)

 The results of GO analysis of 280 DEPs are shown in Fig. 2a. In terms of biological pathways, the DEPs were mainly involved in metabolic processes, cell transformation processes, and single organism metabolic processes. In terms of cell composition, the differentially expressed proteins were mainly located in the cell membrane, organelle, molecular complex and other parts. In terms of molecular functions, the most abundant functions of the DEPs were catalytic activity, binding activity, transport activity, and enzyme regulation activity. The KEGG pathway analysis results are shown in Fig. 2b, which showed that the DEPs were mainly enriched in the cytokine-cytokine receptor interaction, notch signaling pathway, lysine biosynthesis, collecting duct acid secretion, proteasome pathway and other related pathways that may affect semen quality.

### Protein–protein interaction (PPI) analysis of DEPs

To determine the DEPs affecting the semen quality of Niangya yaks, we constructed a PPI network using STRING online software (Additional file 5: Table S4). In this study, the interaction of proteins involved in the significant pathways of the KEGG enrichment analysis was visualized (the nodes in the graph are proteins, and the edges are interactions, the interaction score used in String for network construction in Additional file 6: Table S5). As shown in Fig. 2c, PSMC5, PSMD8, PSMB3, HSP90AA1, UGP2, and HSPB1 were important nodes. Therefore, in the present study we identified 6 candidate proteins which affect the sperm quality in Niangya yaks (Table 2).

**Table 2:** Candidate proteins related to the semen quality of Niangya yaks

Symbol	UniProt ID	GI	Functional classification	Fold change	Regulation
HSPB1	L8IQW1	2236062986	signal passage	0.68	Down
PSMC5	L8HM36	27806101	protein catabolism process	1.55	Up
PSMD8	L8HVD5	555993092	protein hydrolysis	1.43	Up
UGP2	L8I7H2	440901488	UDP-glucose metabolism process	2.09	Up
PSMB3	L8IEX9	440904222	Proteasome-mediated ubiquitin-dependent protein catabolism	1.57	Up
HSP90AA1	L8I2H5	440899031	unfolded protein response	1.62	Up

Fold change is H/L ratio. H = high-quality group (*n* = 3); L = low-quality group (*n* = 3)

### Verification of DEPs by PRM

As shown in Table 3, The PRM analysis results of the 15 DEPs (Additional file 7: Table S6) selected from the TMT were consistent with the analysis results of the TMT-labeled quantitative proteomics.

**Table 3:** Differentially expressed proteins in TMT verified by PRM

Symbol	UniProt ID	GI	TMT-FC	TMT-*P* value	PRM-FC	PRM *P* value	Regulation
HSPB1	L8IQW1	942046753	0.68	0.00	0.42	0.01	Down
PSMC5	L8HM36	440890860	1.55	0.00	1.63	0.05	Up
HSP90AB1	L8IAK0	440908439	1.39	0.00	1.20	0.33	Up
PSMD8	L8HVD5	555993092	1.43	0.00	1.45	0.29	Up
PSMD2	L8HQZ9	440894961	1.39	0.00	1.42	0.21	Up
NSF	L8HXE2	555988346	1.41	0.00	1.89	0.06	Up
PSMD1	L8J565	440913259	1.33	0.00	1.61	0.19	Up
PSMB6	L8I0N4	440897065	1.50	0.00	1.33	0.28	Up
UGP2	L8I7H2	440901488	2.09	0.00	1.82	0.01	Up
PSMB3	L8IEX9	440904222	1.57	0.01	1.55	0.01	Up
HSP90AA1	L8I2H5	440899031	1.62	0.01	1.84	0.07	Up
PSMA4	L8J0V2	440912779	1.54	0.00	1.40	0.06	Up
PSMD12	L8J4A0	555951267	1.42	0.00	1.24	0.35	Up
PSMA3	L8IC20	440901860	1.42	0.00	1.37	0.2	Up
ATP6V1D	L8HL48	440888806	1.45	0.00	1.68	0.06	Up

FC is the fold change of H/L ratio. H = high-quality group (*n* = 3); L = low-quality group (*n* = 3)

## Discussion

The quality of semen is the main factor affecting the conception rate in artificial insemination. Low-motility sperm will affect the conception rate to a certain extent, mainly because low-motility sperm do not typically survive before meeting the egg, or the embryo develops abnormally or dies after fertilization. Sperm with high motility can maintain a high fertilization capability for a long time, which is a prerequisite for improving the conception rate [17], and the level of sperm motility is a key factor in assessing semen quality [18]. Therefore, it is very important to determine the quality of male yak semen [19]. Improving the overall quality of yak semen quality will improve yak reproductive performance and is an important way to improve the yield and economic benefits of yaks [20].

TMT-based quantitative proteomics analysis reveals that the protein expression level of SOD1 protein in the frozen group was significantly lower than that in the fresh group, and the protein expression level of NDUFS8 protein was significantly higher in frozen group [21]. Proteins such as OAZ3, GPx4, and GSTM3 whose upregulation leads to reduced motility were upregulated in the spermatozoa of high ERR bulls, the regulation of ACE, a negative regulator of sperm motility was upregulated in both the spermatozoa and seminal plasma of high ERR bulls [22]. HSPA5 could regulate sperm capacitation and slow down apoptosis by comparative proteomic identification of capacitated and non-capacitated sperm of Yanbian Yellow Cattle [23]. The results of these studies are not quite the same as ours, possibly because of differences in test conditions, species, etc.

PSMD8 has been reported to interact directly with the proteasome-associated deubiquitinase UCH37 [24]. PSMD8 activity may be a rate-limiting factor in polysperm control during fertilization in pigs. Blocking the proteasome subunit PSMD8 increases the rate of sperm passage through the zona pellucida during fertilization in pigs [25]. PSMD8 is an important protein that affects the process of sperm fertilization. Uridine diphosphate glucose pyrophosphorylase (UGPase) is a very important enzyme that produces UDP glucose and is responsible for a key part of carbohydrate biosynthesis in all cells, including the synthesis of sucrose, cellulose, starch, glycogen, and a key component of protein glycosylation and other functions [26]. The enzyme mainly exists as a cytoplasmic soluble protein, and membrane-bound UGPase activity has also been reported in animal and plant tissues [27]. Membrane-bound UGPase activity has also been studied in animal and plant tissues [28]. At the enzyme level, UGPase is only regulated by substrates [29], but it has also been reported that it may constitute a rate-controlling step in sucrose synthesis [30]. UGPase has been found in animals and has been isolated and purified. In mammalian cells, UGPase plays a very important role in metabolic function [31]. These results are also basically the same as ours. In our study UGP2 was upregulated in the high motility sperm group and was mainly involved in the metabolic process. It may affect sperm motility through metabolic processes and have a certain impact on the reproductive ability of yak sperm. These results are also similar to the present findings.

The HSPB1 protein is up regulated in high quality semen group than the low quality. Its protein in the family of small heat-shock proteins (sHSPs), is a key mediator of PRL-mediated inhibition of β-cell apoptosis [32, 33]. A comprehensive protein that can control cellular stress, regulate cellular differentiation and development, and inhibit cancer cells is characterized by a highly conserved α-crystallin domain [34]. sHSPS usually exist in monomeric form, but under certain conditions, they interact to form oligomeric complexes. Some of these chaperones have been shown to play critical roles in a variety of cell types to maintain tissue integrity and function, especially nerve and muscle tissue [35, 36]. Heat shock proteins are not only important for sperm development but are also closely related to epididymal maturation in males and the transformation of sperm function occurring in the female reproductive tract. In many cases of male subfertility, targeted protein disruption of HSPs prevents spermatogenesis, impairs sperm maturation, and inhibits fertilization. Studies have shown that heat shock proteins play an important role in male fertility [37]. The present findings shown that HSPB1 and HSP90AA1 can degrade their target proteins through the ubiquitin‒proteasome system and participate in the regulation of proteasome degradation pathways, signaling and other pathways, especially HSPB1, which plays an important role in improving the overall ubiquitination, folding and degradation of proteins.

In our study, PSMC5 (proteasome26S subunit, ATPase5) ubiquitinates intracellular proteins and participates in protein processing, translocation and degradation. Analysis of DEPs by GO and KEGG showed that PSMC5 in Niangya yak spermatozoa was mainly involved in protein catabolism and had a certain impact on semen quality. PSMC family belongs to the AAA family, which includes 6 ATPases, which can directly affect the metabolism of amino acids and proteins and play an important role in the ATP-dependent ubiquitinated protein degradation process. Most of the genes in the family are involved in processes such as cell cycle regulation, apoptosis, gene expression, and vesicle-mediated transport [38]. This results are basically the same as ours.

## Conclusions

To summarize, in this study, we compared the protein differences between the high- and low-quality sperm of Niangya yaks by TMT labeling quantitative proteomics analysis, screened out the related proteins affecting the semen quality of Niangya yaks, and 15 DEPs were further verified by PRM. We obtained 288 DEPs, of which 6 proteins (PSMC5, PSMD8, PSMB3, HSP90AA1, UGP2 and HSPB1) that were mainly concentrated in energy production and metabolism might. That is, HSPB1, PSMB3 and HSP90AA1 can degrade their target proteins through the ubiquitin‒proteasome system and participate in the regulation of proteasome degradation pathways, signaling and other pathways. The protein PSMC5 is mainly involved in protein catabolism and had a certain impact on semen quality. PSMD8 is an important protein that affects the process of sperm fertilization. UGP2 was upregulated in the high motility sperm group and was mainly involved in the UDP-glucose metabolism process. It may affect sperm motility through metabolic processes and have a certain impact on the reproductive ability of yak sperm. In conclusion, the identified 6 proteins may play important roles in the semen quality of Niangya yaks, which could serve as candidates for the selection and breeding of Niangya yaks.

### Supplementary Information

**Additional file 1: Table S1.** Relevant ion information used for PRM analysis.

**Additional file 2: Fig. S1.** The SDS-PAGE electrophoresis diagram. Note: H-Sperm protein in the high-quality group, L-Sperm protein in the low-quality group.

**Additional file 3: Table S2.** A list of all identified proteins.

**Additional file 4: Table S3.** Details of the differentially expressed proteins.

**Additional file 5: Table S4.** The PPI relationships and scores.

**Additional file 6: Table S5.** The interaction score used in String for network construction.

**Additional file 7: Table S6.** The details of 15 differentially expressed proteins in parallel reaction monitoring.

## Data Availability

All data generated or analyzed during this study are included in this published article and its supplementary information files.

## Abbreviations

TMT: Tandem mass tag
PRM: Parallel reaction monitoring
CASA: Computer-assisted semen analysis
VAP: Average path velocity
VSL: Straight line velocity
VCL: Curvilinear velocity
DTT: Dithiothreitol
LC‒MS/MS: Liquid chromatography-tandem mass spectrometry
FASP: Filter-assisted sample preparation
GO: Gene Ontology
KEGG: Kyoto Encyclopedia of Genes and Genomes
PPI: Protein‒protein interaction
SDS‒PAGE: Sodium dodecyl sulfate‒polyacrylamide gel electrophoresis
