# Supplementary material for: Quantitative proteomics analysis reveals the key proteins related to semen quality in Niangya yaks
Source: Proteome Sci. 2023 Oct 24;21:20. doi: 10.1186/s12953-023-00222-9 (PMC10594827; doi:10.1186/s12953-023-00222-9)
Supplement: Supplementary file 1 — Additional file 1: Table S1. Relevant ion information used for PRM analysis. [file 12953_2023_222_MOESM1_ESM.docx]

Table S1 Relevant ion information used for PRM analysis

| ProteinID | Peptide | M/Z | Z |
| --- | --- | --- | --- |
| L8IQW1 | SATQSAEITIPVTFQAR | 910.481065 | 2 |
| L8IG69 | QTYFLPVIGLVDAEK | 846.963988 | 2 |
| L8HM36 | AVAHHTDCTFIR | 476.566465 | 3 |
| L8IGJ5 | VVIDAFR | 410.239795 | 2 |
| L8ISP4 | LGEYGFQNALIVR | 740.401358 | 2 |
| L8HVD5 | ILFFNTPK | 490.284203 | 2 |
| L8HQZ9 | AVPLALALISVSNPR | 760.961583 | 2 |
| L8HXE2 | TPLVSVLLEGPPHSGK | 544.310278 | 3 |
| L8J565 | AAVESLGFILFR | 661.876987 | 2 |
| L8I0N4 | LAAIAEPGVER | 563.316763 | 2 |
| L8I7H2 | TLDGGLNVIQLETAVGAAIK | 992.059679 | 2 |
| L8IEX9 | LYIGLAGLATDVQTVAQR | 630.354547 | 3 |
| L8I2H5 | ALLFVPR | 408.260531 | 2 |
| L8J0V2 | SALALAIK | 393.757821 | 2 |
| L8J4A0 | DPNNLLNDWSQK | 722.34678 | 2 |
| L8IAK0 | HLEINPDHPIVETLR | 594.988087 | 3 |
| L8IC20 | SNFGYNIPLK | 576.806031 | 2 |
| L8IKU9 | AIHSWLTR | 492.274701 | 2 |
| L8HP74 | EVATNSELVQSGK | 681.348988 | 2 |
| L8HL48 | EAAFSLAEAK | 518.769114 | 2 |
