# Supplementary figures and images for: Quantitative proteomics analysis reveals the key proteins related to semen quality in Niangya yaks
Source: Proteome Sci. 2023 Oct 24;21:20. doi: 10.1186/s12953-023-00222-9 (PMC10594827; doi:10.1186/s12953-023-00222-9)

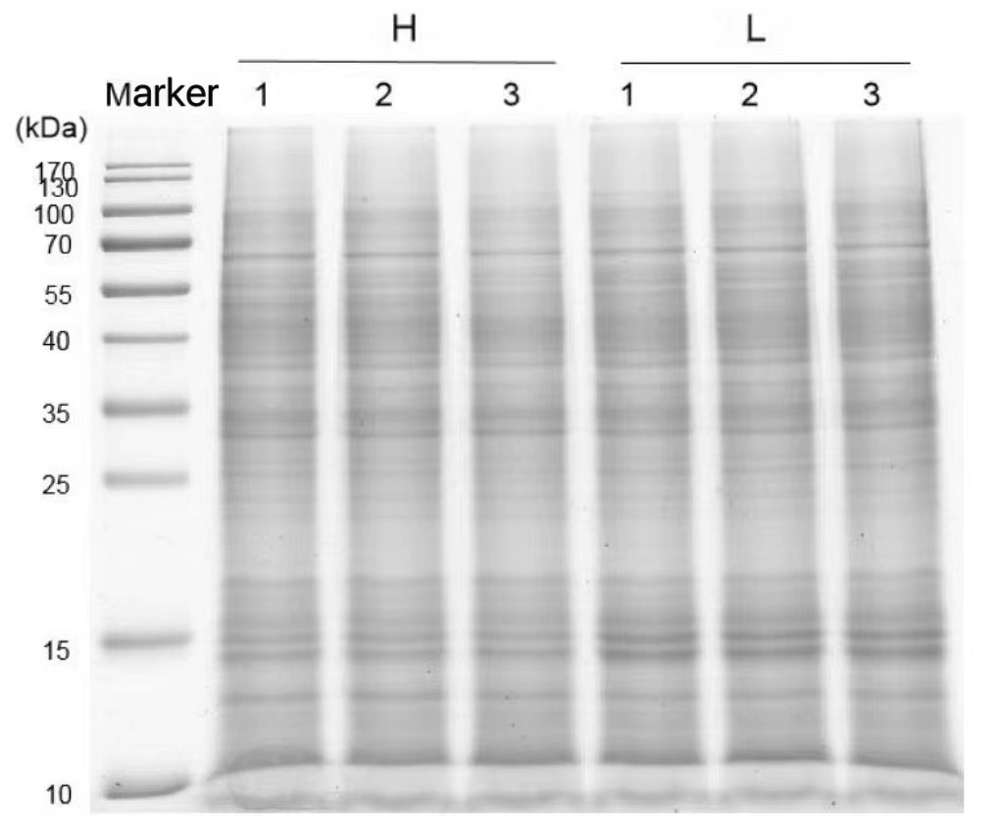

Supplement: Supplementary file 2 — Additional file 2: Fig. S1. The SDS-PAGE electrophoresis diagram. Note: H-Sperm protein in the high-quality group, L-Sperm protein in the low-quality group. [file 12953_2023_222_MOESM2_ESM.jpg]
